# Toxoplasma gondii retinochoroiditis associated with Epstein-Barr virus-induced retinal necrosis in an immunocompetent patient

**DOI:** 10.1186/s12348-026-00611-z

**Published:** 2026-06-11

**Authors:** Maria Carmela Saturno, Danilo Iannetta, Ngoc Chau Nguyen, Lorette Leon, Marc D. de Smet

**Affiliations:** 1 Helvetia Retina Associates, MIOS sa, Lausanne, Switzerland; 2 Department of Sense Organs, Sapienza University of Rome, Rome, Italy; 3 Centre Neuchâtelois d’Ophtalmologie, Neuchâtel, Switzerland; 4 New York Eye and Ear Infirmary of Mt Sinai, Icahn School of Medicine, New York City, NY USA

**Keywords:** Retinochoroiditis, Retinal necrosis, Toxoplasma gondii, Epstein–Barr virus, Methotrexate, Intravitreal therapy

## Abstract

**Purpose:**

To describe a rare case of concomitant Toxoplasma gondii (Tg) retinochoroiditis and Epstein–Barr virus (EBV) retinal necrosis in an immunocompetent patient, successfully treated with a combined intravitreal and systemic therapeutic approach.

**Methods:**

A 52-year-old immunocompetent woman with recurrent bilateral Tg retinochoroiditis presented with reactivation in the left eye unresponsive to standard antiparasitic therapy. Comprehensive ophthalmological examination, including diagnostic pars plana vitrectomy with vitreous biopsy and polymerase chain reaction (PCR) analysis, was performed.

**Results:**

PCR analysis of the vitreous sample confirmed the presence of both Tg and EBV. Intravitreal methotrexate (MTX) was initiated and administered over an 8-week period in combination with ongoing intravitreal clindamycin and systemic antiparasitic therapy, together with systemic acyclovir (800 mg three times daily). The patient demonstrated progressive clinical improvement, with stabilization of visual acuity and retinal morphology on optical coherence tomography (OCT). Serial vitreous PCR monitoring revealed a gradual decline in Tg and EBV loads until both pathogens became undetectable. No recurrence occurred during follow-up.

**Conclusion:**

This report describes a rare case of Tg–EBV coinfection–related retinochoroiditis in an immunocompetent patient. The favorable outcome observed after initiation of intravitreal MTX, in combination with systemic acyclovir, suggests that MTX may have contributed to disease control through immunomodulatory suppression of B-cell proliferation and possible antiviral effects. This case underscores the importance of considering coinfection in atypical or treatment-refractory retinochoroiditis, even in immunocompetent hosts.

## Introduction

Toxoplasma gondii (Tg) is a ubiquitous intracellular parasite, with approximately one-third of the global human population estimated to have been exposed, representing one of the leading causes of posterior uveitis [1, 2]. The classic presentation is a unilateral, unifocal retinochoroiditis with overlying vitritis, although milder infections may manifest as multiple small outer retinal opacities, known as “punctate outer retinal toxoplasmosis”. Persistent or reactivated infection, particularly in immunocompromised hosts, may lead to extensive retinal necrosis mimicking other necrotizing retinitis, such as cytomegalovirus retinitis. Severe or atypical cases can also occur in elderly or immunocompetent patients infected with virulent strains [1–4]. The diagnosis of ocular toxoplasmosis (OT) is mainly clinical, but biological confirmation is crucial in atypical or treatment-resistant cases. Despite its incidence, the optimal therapy for OT is controversial and there is no consensus on which drugs must be used for prophylactic treatment of recurrences [1]. Coinfections with other pathogens, such as Epstein-Barr virus (EBV), have been reported in 7–8% of patients, regardless of immune status, complicating diagnosis and it is linked to a worse prognosis [4].

EBV is a ubiquitous DNA virus of the Herpesviridae family infecting over 90% of humans. It primarily targets B lymphocytes, establishing lifelong latency within these cells. Although usually asymptomatic, EBV can cause systemic and ocular diseases, including necrotizing retinitis, especially in immunocompromised individuals. In terms of treatment, antivirals or corticosteroids have shown variable benefits in isolated reports. In 2008, Frenkel et al. reported favorable outcomes describing a 10-year experience with intravitreal methotrexate (IVT MTX) for the treatment of vitreoretinal involvement in primary central nervous system lymphoma. As an antifolate antimetabolite, MTX inhibits DNA synthesis and suppresses the proliferation of activated B lymphocytes, the main reservoir of EBV. Based on this mechanism, IVT MTX has shown efficacy in EBV-related vitreoretinal lymphoma, and in few reported EBV-associated retinal necrosis, either alone or in combination with antiviral agents [5–9].

We hereby report a rare case of coinfection by EBV and Tg in an immunocompetent patient presenting with retinochoroiditis successfully treated by a combination of systemic antiviral and antiparasitic therapy, as well as IVT clindamycin and MTX.

## Case presentation

A 52-year-old immunocompetent woman with a history of depression, treated with venlafaxine, mirtazapine and pregabalin, had been followed at Helvetia Retina Associates, MIOS sa, Lausanne, Switzerland for bilateral Tg retinochoroiditis since 2009. The left eye (LE) experienced multiple recurrences from 2014 and underwent cataract surgery in 2023, achieving a postoperative best-corrected visual acuity (BCVA) of 20/20.

In April 2024, a further recurrence in the LE was treated with oral azithromycin (500 mg on day 1, followed by 250 mg daily) and oral prednisone at a starting dose of 80 mg/day, which was gradually tapered. Given multiple risk factors for recurrence, including age over 40 years, bilateral disease, macular involvement and extensive lesion size, a six-month prophylactic regimen with pyrimethamine 25 mg and folic acid 15 mg was initiated in August 2024.

However, prophylaxis was interrupted during a hospital admission for severe depression and the patient presented in October 2024 with recurrent inflammation in the LE. On presentation, BCVA had decreased to 20/60 and slit-lamp examination revealed 3 + anterior chamber cells, 1 + flare, vitritis and IOP was 43 mmHg. She was treated with azithromycin (500 mg on day 1 and 250 mg daily), topical dexamethasone (Dexafree^®^) six times daily, topical brinzolamide-brimonidine (Simbrinza^®^) twice daily and oral acetazolamide 250 mg twice daily, resulting in progressive normalization of intraocular pressure. Because of intolerance to systemic corticosteroids, causing insomnia and mood deterioration, a topical approach was preferred and an IVT implant of dexamethasone 700 µg (Ozurdex^®^) was administered in her LE, resulting in temporary control of inflammation and visual improvement.

**Fig. 1:**
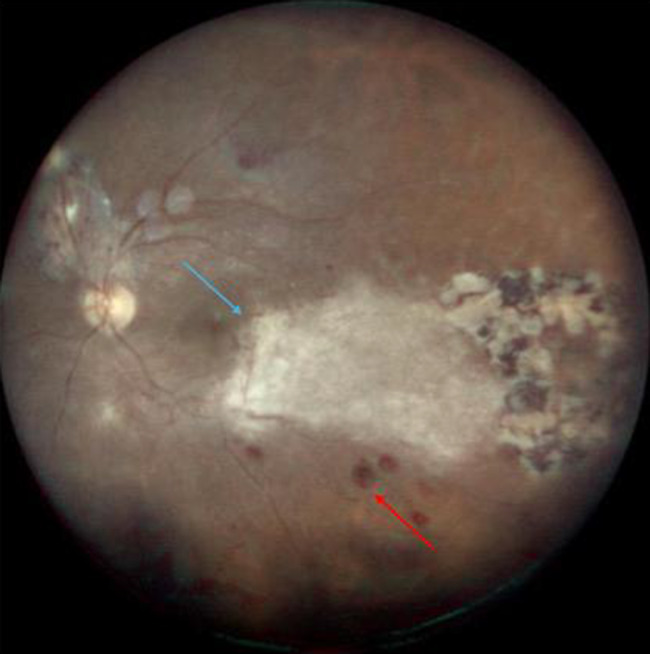
Wide-field color fundus photograph of the left eye showing a large area of retinal infiltration with a sharp leading edge extending temporally to the fovea. Perivascular sheathing of adjacent veins and arterioles is visible, along with scattered blot hemorrhages in the surrounding retina. Blue arrow indicates the sharply demarcated leading edge of retinal infiltration, while red arrows indicate adjacent blot hemorrhages

**Fig. 2:**
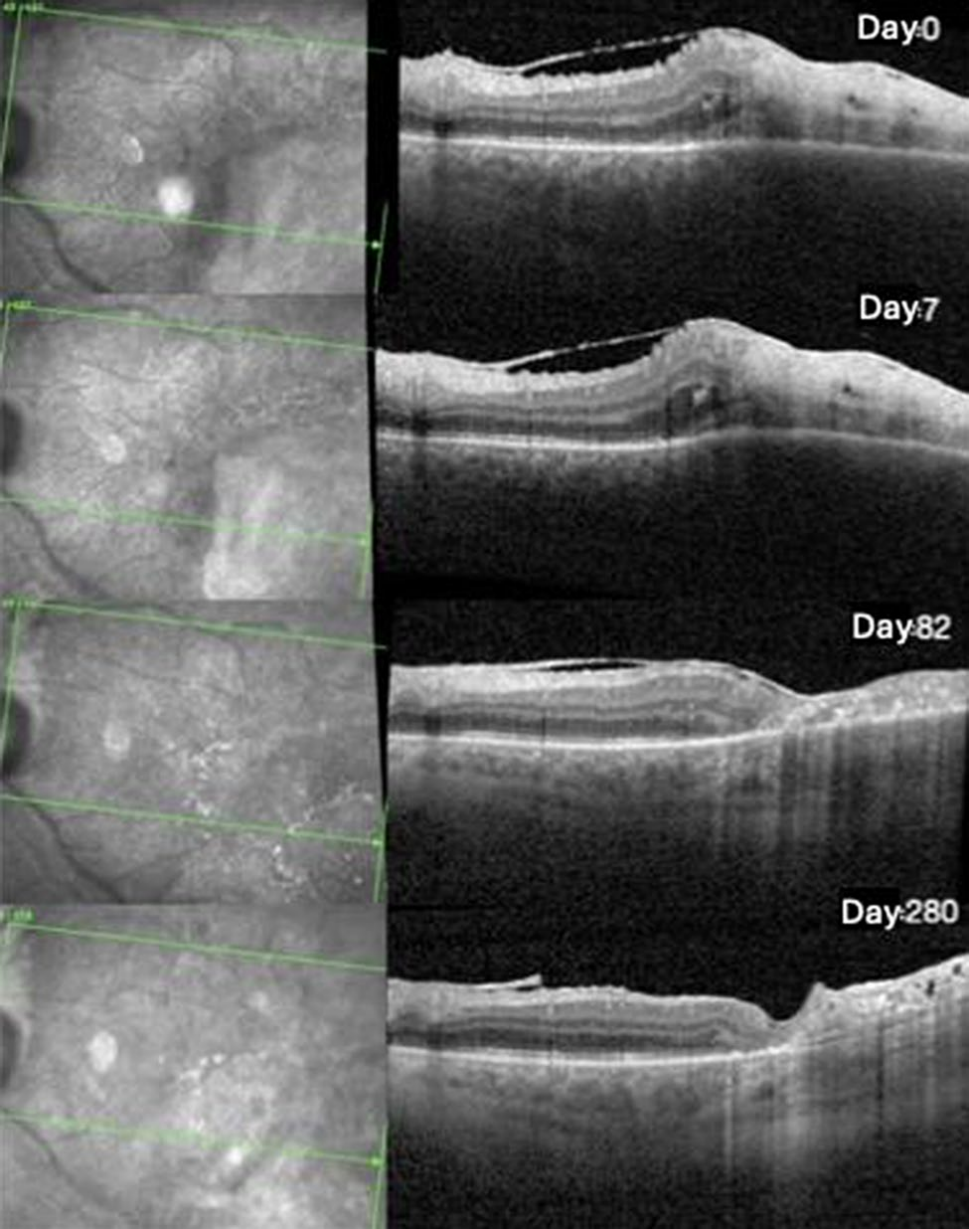
Serial spectral-domain optical coherence tomography (OCT) scans through the fovea obtained at day 0, day 7, day 82 and day 280 after initiation of therapy. The sequence illustrates the evolution of the lesion, showing absence of further central progression and a gradual structural improvement at the margins of the fovea over time. The last image shows no epiretinal membrane, as it had been surgically peeled beforehand

In January 2025, during a follow up visit, the patient referred blurred vision. The ophthalmological examination revealed BCVA declined to 20/33 and a large area of retinal infiltration with a sharp leading edge to the temporal side of the fovea. Sheathing of neighbouring veins and arterioles, as well as blot hemorrhages were also noted in the adjacent retina (Fig. 1). Because the patient admitted discontinuing prophylactic treatment during Christmas holidays, a further reactivation of Tg was suspected, and oral trimethoprim–sulfamethoxazole (Bactrim forte^®^) twice daily was initiated. Despite the medical treatment, the clinical course worsened, with progressive visual decline and enlargement of the retinal lesion. Optical coherence tomography (OCT) revealed marked retinal thickening, retinal hyperreflectivity and loss of retinal stratification, without choroidal involvement, vitreous cells or preretinal deposits (Fig. 2, day 0). Given the atypical clinical and imaging features, and after failing to achieve disease control with a single intravitreal injection of clindamycin, a pars plana vitrectomy with vitreous biopsy and removal of the Ozurdex^®^ implant was performed.

Polymerase chain reaction (PCR) analysis of the vitreous sample was positive for both Tg and EBV. Based on these findings, IVT MTX 400 µg was initiated in combination with IVT clindamycin (1 mg), systemic acyclovir (800 mg three times a day) and Bactrim forte^®^ (twice daily). Nine injections spaced between 5 and 14 days were given, based on the clinical response and PCR data (Table 1). MTX injections were continued until the EBV titers were negative on two repeat measurements. Clindamycin was discontinued when the Tg lesion appeared clinically quiescent.

**Table 1:** Treatment and response characteristics following the initiation of intravitreal methotrexate

Days since IVT initiation	Visual acuity	Quantitative PCR Titers		Intravitreal Therapy		Systemic Therapy **	
		EBV	Toxoplasmosis	Methotrexate	Clindamycin*	Acyclovir	Sulfamethoxazole + trimethoprim
**0**	0.12	28,229	254,757	x	x	x	x
**5**	0.2	1211	20,522	x	x	x	x
**12**	0.2	1091	7937	x	x	x	x
**20**		800	2177	x	x	x	x
**26**	0.2	928	2620	x	x	x	x
**33**	0.13	1022	1046	x	x	x	x
**41**	0.2	0	485	x	x	x	x
**54**	0.12	0	141	x		x	x
**160**	0.16	0	0				x
**280**	0.2						

Visual acuity values are reported in decimal notation

Symbol “x” indicates that treatment was administered

EBV: Epstein-Barr virus; IVT: intravitreal therapy

* IVT Clindamycin was given once, prior to the IVT methotrexate one week prior at the end of the vitrectomy

** For systemic therapy, the symbol “x” indicates that treatment was ongoing at that time point. Oral trimethoprim–sulfamethoxazole was switched to pyrimethamine and folic acid on day 160

**Fig. 3:**
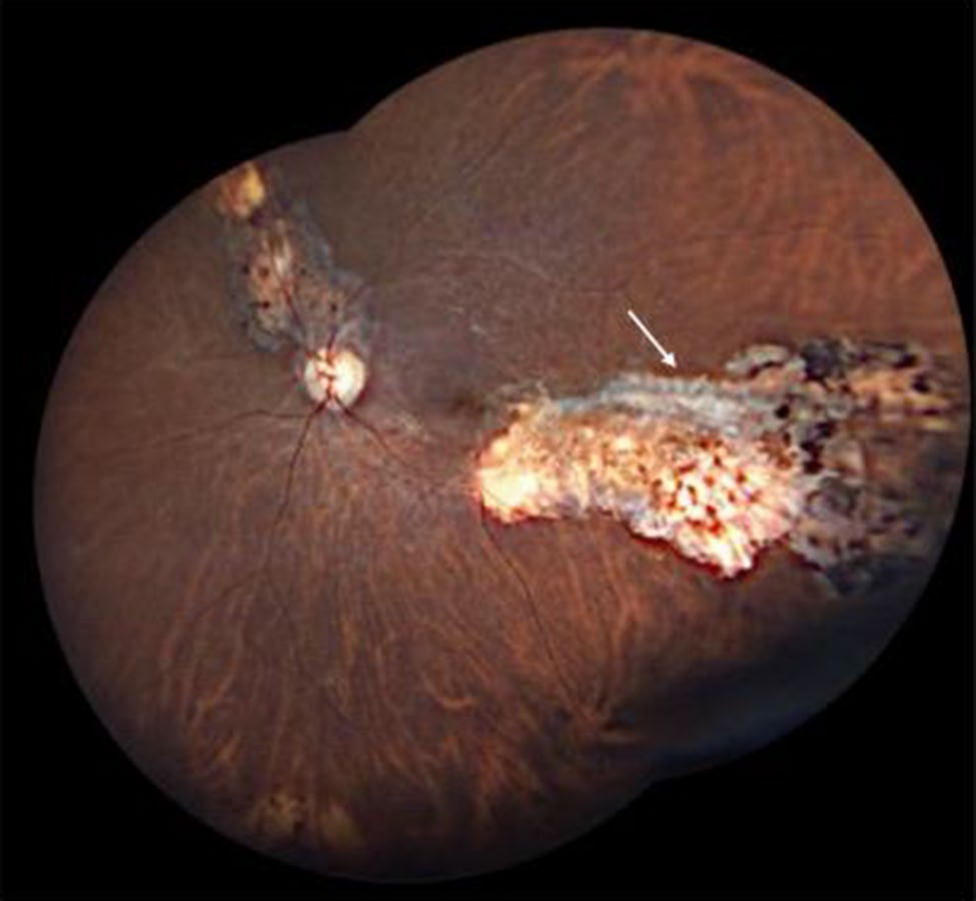
Wide-field color fundus photograph obtained nine months after initiation of therapy showing the healed area of paramacular retinitis. The lesion is characterized by retinal pigment epithelium (RPE) atrophy with sparse pigmentation, distinct from the more pigmented toxoplasmosis scars. A white arrow highlights areas of mild fibrosis along the borders of the scar. The epiretinal membrane previously present over the fovea had surgically peeled

Over subsequent weeks, intraocular inflammation progressively subsided, and no further extension of the retinal lesion was observed on widefield imaging or OCT (Fig. 2, day 280). Serial vitreous PCR demonstrated a gradual decline in both EBV DNA and Tg loads. BCVA improved to 20/25 and remained stable at the most recent follow-up. Nine months after treatment initiation, the area of EBV-associated paramacular retinitis evolved into retinal pigment epithelium atrophy with minimal pigmentation and localized fibrosis; a coexisting epiretinal membrane was surgically peeled in July 2025 (Fig. 3).

## Discussion

Concomitant Tg and EBV retinochoroiditis is exceedingly rare, particularly in immunocompetent individuals [10]. A similar coinfection has been described by Mejìa-Salgado et al., in a pharmacologically immunosuppressed patient receiving corticosteroids, MTX and abatacept for rheumatoid arthritis. In their report, the patient developed bilateral granulomatous anterior chamber inflammation, vitritis and extensive retinal necrosis with macular involvement. The concomitant infection was confirmed by PCR analysis of vitreous samples and the patient was treated with combined oral antiviral and antiparasitic therapy [2]. In contrast, our patient achieved sustained remission within two months through a combined pathogen-directed strategy consisting of IVT MTX together with systemic acyclovir for EBV, as well as IVT clindamycin and oral trimethoprim–sulfamethoxazole for Tg. Although serial PCR demonstrated progressive EBV clearance after MTX initiation, the concomitant use of systemic acyclovir precludes attributing the antiviral effect exclusively to MTX.

Moreover, our case underscores the diagnostic challenges posed by rare ocular coinfections, particularly in patients with a known history of recurrent Tg retinochoroiditis, in whom disease reactivation may be erroneously attributed to Tg alone. In this context, OCT plays a pivotal role in raising suspicion for an alternative or additional etiology. As described by Pichi et al., Tg retinochoroiditis typically presents with hyperreflective preretinal deposits with associated choroidal involvement, whereas viral retinitis predominantly affects only retinal layers without choroidal infiltration [11]. In our patient, the emergence of a new retinal lesion characterized by marked retinal thickness and hyperreflectivity on OCT, in the absence of choroidal or preretinal involvement (Fig. 2, day 0), together with a poor response to conventional antiparasitic therapy, raised suspicion for an additional infectious etiology. The recent administration of an IVT dexamethasone implant may also have contributed to the atypical presentation and rapid progression observed in this case, as local corticosteroid-induced immunosuppression is a recognized risk factor for the reactivation or worsening of both viral retinitis and ocular toxoplasmosis [3]. Subsequently, vitreous biopsy under air [12] and PCR played a crucial role in establishing the definitive diagnosis.

Furthermore, quantitative PCR on serial vitreous samples proved valuable in guiding targeted therapy, monitoring treatment response and allowing discontinuation of IVT therapy in only two months, when the titers were at the level of detection or below [6, 7].

From a therapeutic standpoint, the management of EBV-related retinitis remains difficult and controversial [6–8]. In vitro studies have shown that anti-herpesvirus drugs such as nucleoside/nucleotide analogs (ganciclovir, cidofovir) or pyrophosphate analogs (foscarnet) inhibit EBV-DNA polymerase activity [9]. However, in most clinical cases a slow rate of response is observed, associated with a significant vision loss [2, 13].

In this context, there is emerging evidence of IVT MTX’s antiviral effects in EBV-associated retinal necrosis [6–8]. MTX inhibits purine and pyrimidine synthesis, suppressing T- and B-cell proliferation and downregulating inflammatory cytokines such as TNF-α, IFN-γ and IL-6 [6–8, 14]. Mashima et al. first described an 83-year-old immunosuppressed woman who developed PCR-confirmed EBV necrotizing retinitis unresponsive to systemic acyclovir and ganciclovir. After 15 IVT MTX injections, both inflammation and EBV DNA load decreased, leading to a clinical improvement [6]. Similarly, Cho et al. reported a renal transplant recipient with EBV-induced necrotizing retinitis confirmed by vitreous PCR. The disease progressed despite IVT ganciclovir and foscarnet, but the addition of IVT MTX resulted in a clinical improvement [8].

Mechanistically, the pathogenesis of EBV-related retinal disease remains poorly defined, particularly regarding how the virus’s established biology culminates in retinal necrosis. Beyond its well-established B-cell tropism, EBV has been implicated in neuroinflammatory and neurodegenerative disorders. Studies suggest that EBV may exert direct neurotropic effects or indirectly promote tissue injury through infected B cells, leading to neuroinflammation, demyelination, glial activation and neuronal degeneration [15, 16]. EBV has been observed within neurons and glial cells of patients affected by multiple sclerosis, with viral burden correlating with disease severity in postmortem brain tissue [17, 18]. By analogy, similar mechanisms are likely present in retinal neural tissue.

Finally, this case also highlights the potential relevance of the patient’s chronic depressive disorder as a contributing factor. Chronic depression has been associated with reduced natural killer cell cytotoxicity, altered B-cell homeostasis, elevated proinflammatory cytokines resulting in an impairment of the innate immune responses and suggesting higher susceptibility to viral infections [19–21].

In conclusion, our report suggests that concomitant Tg and EBV infection may occur in immunocompetent individuals. Intravitreal therapy with MTX, administered in combination with systemic antiviral treatment, may contribute to rapid reduction in intravitreal EBV titers, and serial quantitative PCR may be useful in monitoring treatment response and guiding therapeutic duration.

## Data Availability

All data generated or analyzed during this study are included in this published article. Additional information is available from the corresponding author upon reasonable request.

## Abbreviations

Tg: Toxoplasma gondii
EBV: Epstein–Barr virus
PCR: Polymerase chain reaction
MTX: Methotrexate
OCT: Optical coherence tomography
OT: Ocular toxoplasmosis
IVT: Intravitreal
BCVA: Best-corrected visual acuity
LE: Left eye
